# Effect of curcumin on glycerol-induced acute kidney injury in rats

**DOI:** 10.1038/s41598-017-10693-4

**Published:** 2017-08-31

**Authors:** Jindao Wu, Xiongxiong Pan, Heling Fu, Yuan Zheng, Youjin Dai, Yuan Yin, Qin Chen, Qingting Hao, Dan Bao, Daorong Hou

**Affiliations:** 1 Key laboratory of Living Donor Liver Transplantation, National Health and Family Planning Commision, Department of Liver Transplantation Center, The first Affiliated Hospital of Nanjing Medical University, 300 Guangzhou Road, Nanjing, 210029 People’s Republic of China; 2 Department of Anesthesiology, First Affiliated Hospital of Nanjing Medical University, 300 Guangzhou Road, Nanjing, 210029 People’s Republic of China; 3 Key laboratory of the model animal, Animal Core Facility of Nanjing Medical University, Nanjing Medical University, 101 Longmian Avenue, Nanjing, 211166 People’s Republic of China

## Abstract

The aim of this study was to investigate the protective role and underlying mechanisms of curcumin on glycerol-induced acute kidney injury (AKI) in rats. Glycerol (10 ml/kg BW, 50% v/v in sterile saline, i.m.) was used to induce AKI, followed by curcumin (200 mg/kg/day, p.o.) administration for 3 days. To confirm renal damage and the effects of curcumin on AKI, serum BUN, Scr, and CK as well as renal SOD, MDA, GSH-Px were measured. Additionally, morphological changes were identified by H&E staining and transmission electron microscopy. The expression of several factors including chemotactic factor MCP-1, proinflammatory cytokines including TNF-α and IL-6, as well as the kidney injury markers, as Kim-1 and Lipocalin-2 were also assessed using q-PCR. Finally, cell apoptosis in renal tissue was detected using *in situ* TUNEL apoptosis fluorescence staining and expression of proteins associated with apoptotic, oxidative stress and lipid oxidative related signaling pathways were detected using immunohistochemical staining and western blot. The results showed that curcumin exerts renoprotective effects by inhibiting oxidative stress in rhabdomyolysis-induced AKI through regulation of the AMPK and Nrf2/HO-1 signaling pathways, and also ameliorated RM-associated renal injury and cell apoptosis by activating the PI3K/Akt pathway.

## Introduction

Acute kidney injury (AKI) is a syndrome characterized by an acute loss of renal function, and is associated with increased mortality, prolonged hospital stays and accelerated chronic kidney disease^1^. AKI is a relatively a newly classified disease first introduced by the emergency medical community and the international society of nephrology^2^, which replaced the concept of traditional acute renal failure (ARF), and was proposed in order to advance clinical diagnosis of the disease^3^. Despite the reversibility of the loss of renal function in most patients who survive, the mortality of AKI remains alarmingly high (over 50%)^3, 4^ and to date, no effective therapies to prevent or treat AKI exist.

Rhabdomyolysis (RM) is defined as a massive breakdown of skeletal muscle in which potentially large of amounts of damaging intracellular content enters into blood^5^. The development of RM is associated with causes such as crush syndrome, exhaustive exercise, medications, infections and toxins^6–9^. AKI is one of the most severe complications of RM, with approximately 15% of patients with RM developing AKI^6^, and 5–15% of AKI cases are attributed to RM^7^. Myoglobin-induced renal toxicity plays a key role in RM-associated AKI by increasing oxidative stress, inflammation, endothelial dysfunction, vasoconstriction, and apoptosis^10^. In recent years, research into effective therapies to prevent and recovery AKI have attracted much attention, although to date, there is no established therapy to promote recovery.

Experimental AKI induced by glycerol injection is the most commonly used experimental model of RM^1, 11, 12^ and is characterized by intense cortical acute tubular necrosis and inflammatory cell infiltration^13^. Curcumin is an active component in turmeric rhizomes (*Curcuma Longa Linn*), and is a polyphenolic curcuminoid that accounts for 3–5% of turmeric^14^ which has been shown to have antioxidant^15–18^, anti-inflammatory^19–21^, anti-apoptotic^22^ and anti-bacterial^23^ functions. The role of curcumin as a protective agent against renal injury induced by gentamicin, contrast agent, cisplatin and diabetes has been previously investigated^24–32^. However, the mechanisms for the anti-apoptotic and antioxidant effects of curcumin on AKI have not been elucidated, nor the effects of curcumin on experimental AKI models of RM. In this study, the protective role and underlying mechanisms of curcumin on glycerol-induced AKI in rats were investigated.

## Results

### Biochemical analysis of the effects of curcumin on serum and renal tissue

**Figure 1:**
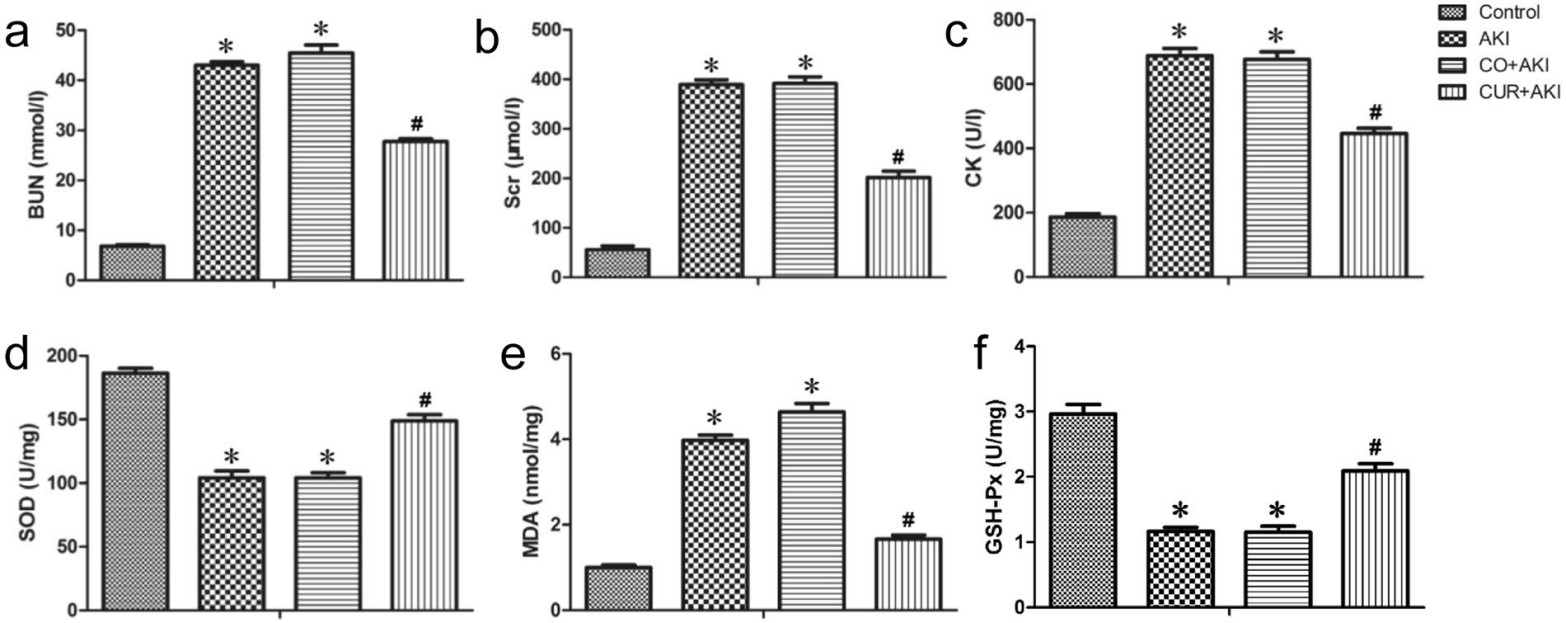
Effect of curcumin on serum BUN, Scr, CK and kidney SOD, MDA and GSH-Px in rats. Curcumin treatment on renal tissue ameliorates **glycerol**-induced acute kidney injury. (**a**,**b**,**c**) Compared with the control group, curcumin significantly reduced Scr, BUN and CK levels 72 h after **glycerol** injection. Statistical significance: *p < 0.01 versus the control group; ^#^p < 0.01 versus the AKI and CO + AKI groups, respectively (n = 12). Data are represented the means ± SD. (**d**,**e**,**f**) Curcumin significantly reduced SOD and GSH-Px levels compared with the control group and markedly increased MDA compared with AKI and CO + AKI groups 72 h after **glycerol** injection. Each bar represents the mean ± SD (n = 12). Statistical significance: *p < 0.01 versus the control group; ^#^p < 0.01 versus the AKI and CO + AKI group.

The effects of curcumin on kidney function and oxidative stress levels in rats are shown in Fig. 1. There was no significant difference in serum urea and creatinine levels between the AKI and CO + AKI (AKI treated with corn oil) groups. Glycerol injection significantly (p < 0.01) increased blood urea nitrogen (BUN), and serum creatinine (Scr) and ceatine kinase (CK) levels in both the AKI and CO + AKI groups compared with the control group (Fig. 1a,b,c). Curcumin treatment resulted in a significant reduction in BUN, Scr and CK levels in CUR + AKI (AKI treated with curcumin) group compared with the AKI and CO + AKI groups, respectively (p < 0.01).

In addition, kidney tissue superoxide dismutase (SOD) enzyme activity and glutathione peroxidase (GSH-Px) enzyme activity levels decreased significantly in the AKI and CO + AKI groups compared with the control group (p < 0.01) (Fig. 1d,f). Kidney tissue MDA levels increased significantly (p < 0.01) in the AKI and CO + AKI groups compared with the control group (p < 0.01) (Fig. 1e). Curcumin treatment resulted in a significant increase in SOD and GSH-Px levels, and a reduction in MDA levels compared with the AKI and CO + AKI groups.

### Effects of curcumin on renal histopathology

**Figure 2:**
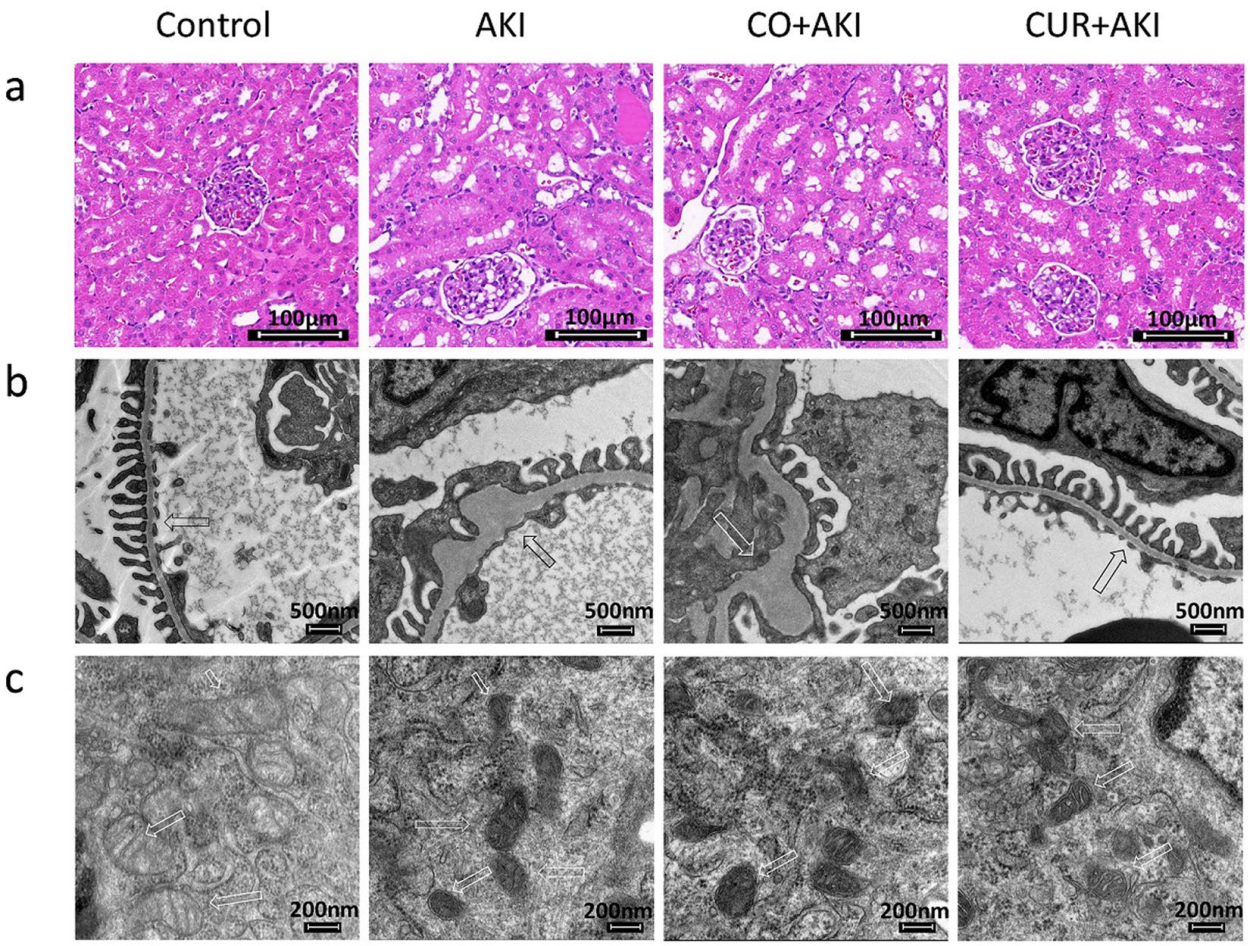
Effect of curcumin on kidney histological morphology changes. (**a**) Histopathological examination of kidney tissue by H&E staining (200×). (**b**,**c**) Representative TEM photomicrographs showing GBM thickness, open slit pores and mitochondrial cristae (arrows). Control: kidney section from normal rats injected with saline; AKI and CO + AKI: Kidney section of AKI rats and AKI rats treated with corn oil showing significant tubular necrosis, cavity expansion, GBM tumefaction, mitochondrial crest loss, and reduced numbers of open slit pores. CUR + AKI: Kidney section of AKI rats treated with curcumin showing significantly improved renal morphology compared to the AKI and CO + AKI groups.

Histopathological appearances in the experimental groups and in the control group are shown in Fig. 2. Kidney sections from control rats displayed normal renal tissue structure, complete renal tubular epithelial cells and no obvious pathological changes in glomerular or renal interstitium (Fig. 2a); kidney tissue from AKI and CO + AKI rats showed kidney tubule cavity expansion and glomerular hypertrophy as well as renal tubular epithelial cell edema and interstitial inflammatory infiltration (Fig. 2a). Curcumin treatment significantly relieved severity of renal lesions and renal tubular injury in the CUR + AKI group. Specifically, tubular injury scores showed that, compared with control group, tubular injury increased in AKI and CO + AKI groups (p < 0.01) (Supplementary Fig. 1), but after curcumin treatment, this increase was signficantly negated (p < 0.01).

The results from TEM showed that, within the AKI and CO + AKI groups, glomerular basement membrane (GBM) thickness was increased and podocyte foot processes were effaced; the number of slit pores between the podocyte foot processes was significantly decreased (Fig. 2b); podocyte mitochondrial cristae were not apparent or disappeared; and endoplasmic reticulum expansion was evident, as were increases in secondary lysosomes (Fig. 2c). In contrast, curcumin treatment significantly ameliorated the overall lesion range. Specfically, GBM thickness was decreased, there was less fading of the podocyte foot processes and the number of open slit pores were increased compared with AKI and CO + AKI groups (Fig. 2b,c).

### Effect of curcumin on renal immunohistochemistry

**Figure 3:**
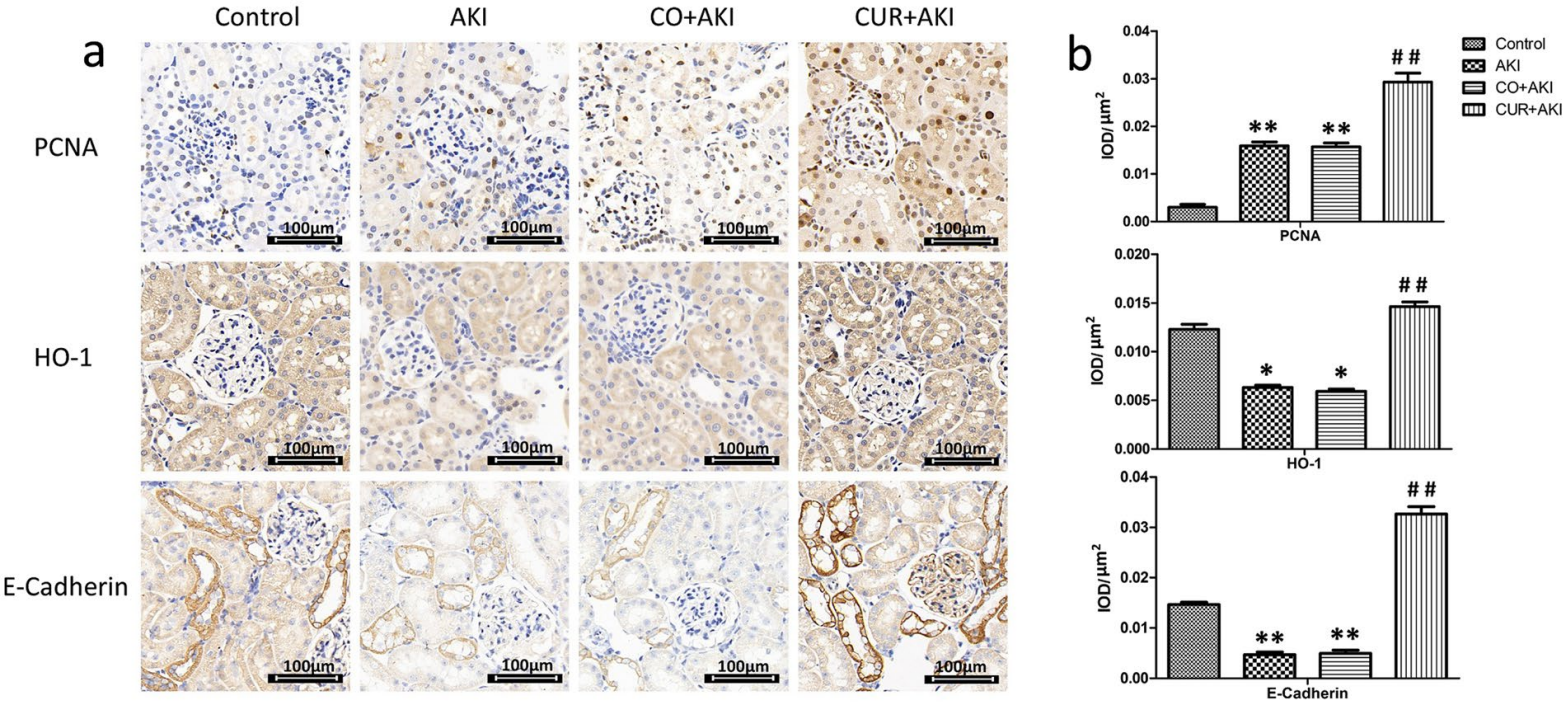
Expression of HO-1, PCNA and E-cadherin in kidney tissue. (**a**) Representative immunohistochemical images of PCNA, HO-1 and E-cadherin in sections of rat kidney from the control group, the AKI group, the CO + AKI group, and the CUR + AKI group. Curcumin treatment significantly increased expression of PCNA, HO-1 and E-cadherin in renal tissue of rats in the CUR + AKI group. (**b**) Semi-quantitative evaluation of PCNA, HO-1 and E-cadherin expression represented as IOD/μm^2^. Each bar represents means ± SD (n = 8). Statistical significance: *p < 0.01 versus the control group; ^#^p < 0.01 versus the AKI and CO + AKI groups, respectively. IOD: integrated optical density.

To further examine the effects of curcumin in the kidney, PCNA, HO-1 and E-cadherin expression were assessed by immunohistochemical staining (Fig. 3a and Supplementary Fig. 2a). Semi-quantitative evaluation of PCNA, HO-1 and E-cadherin expression in renal tissue were considered as the total expression of these proteins in the nucleus and cytoplasm (Fig. 3b and Supplementary Figure 2b). PCNA expression was observed in proliferating cell nuclei, and was significantly increased in AKI and CO + AKI groups compared with the control group (p < 0.01). In control group, PCNA-positive staining was weak in the kidney sections while there was a higher level of expression of PCNA in the CUR + AKI group compared with the AKI and CO + AKI groups (p < 0.01). In addition, HO-1 expression was down-regulated in AKI and CO + AKI groups compared with control group (p < 0.05); intense HO-1 staining was observed in the renal tubular epithelial cell cytoplasm in CUR + AKI group, levels were higher than that of the AKI and CO + AKI groups (p < 0.01). E-cadherin expression was also observed in tubular segments epithelial cells and significantly decreased in the AKI and CO + AKI groups compared with control group (p < 0.01). Finally, there was higher expression of E-cadherin in the CUR + AKI group compared with AKI and CO + AKI groups (p < 0.01).

### Effects of curcumin on glycerol-induced renal apoptosis

**Figure 4:**
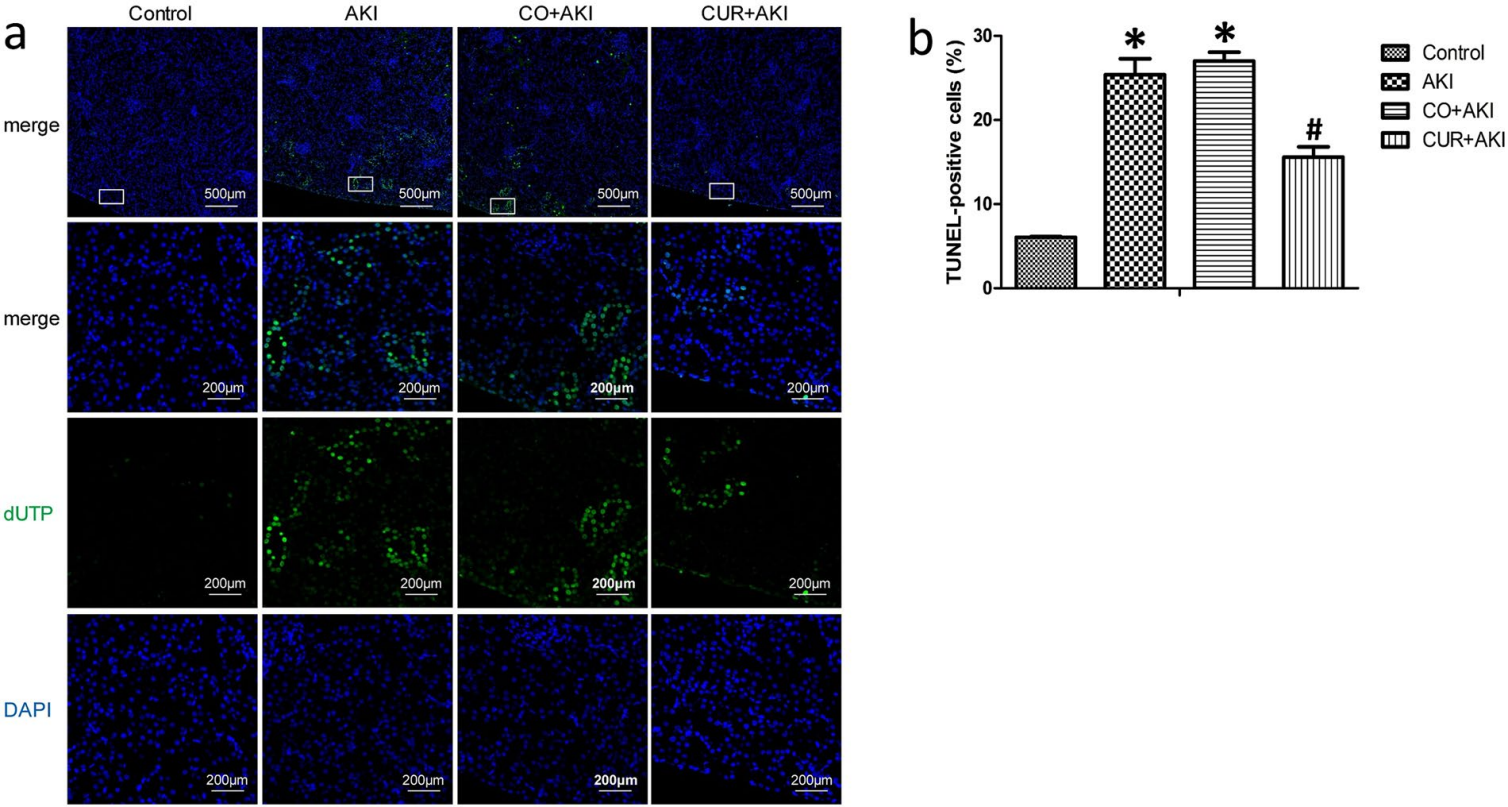
Effect of curcumin on glycerol-induced renal apoptosis. (**a**) Representative *in situ* TUNEL fluorescence images in sections of rat kidney from the control group, the AKI group, the CO + AKI group and the CUR + AKI group. The AKI and CO + AKI groups displayed markedly increased numbers of TUNEL-positive cells than that in the control group, mainly in the tubular area of renal cortex. Curcumin treatment decreased the number of TUNEL-positive cells, and fewer apoptotic cells were observed. (**b**) The levels of apoptosis were indicated as the percentage of TUNEL-positive cells among total cells. Five random fields per section were examined in each experiment. Each bar represents means ± SD (n = 8). Statistical significance: ^*^p < 0.01 versus the control group; ^#^p < 0.01 versus the AKI and CO + AKI groups, respectively.

In the TUNEL assay, the nuclei of TUNEL-positive cells were stained green, indicating apoptotic cells (Fig. 4a and Supplementary Fig. 3a). The levels of apoptosis were indicated as the percentage of TUNEL-positive cells among total cells (Fig. 4b and Supplementary Fig. 3b). TUNEL-positive cells were observed mainly in the tubular area of the renal cortex, although some TUNEL-positive cells were detached from the tubular basement membrane in the lumen. Few apoptotic cells were observed in the control group, whereas the AKI and CO + AKI groups displayed more TUNEL-positive cells than the control group (p < 0.01). As expected, curcumin treatment decreased the number of TUNEL-positive cells significantly, and fewer apoptotic cells were observed in the CUR + AKI group compared with the AKI and CO + AKI groups (p < 0.01).

### Effects of curcumin on the mRNA expression of inflammation and kidney injury markers in renal tissue

**Figure 5:**
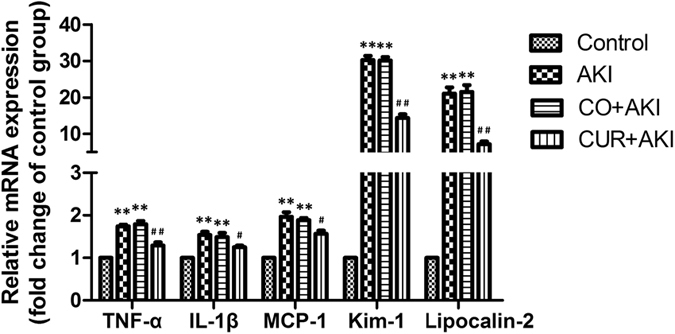
Effect of curcumin on mRNA expression of inflammatory markers and kidney injury markers in kidney tissue. Relative expression (fold) of TNF-α, IL-6, MCP-1 Kim-1 and Lcn-2 mRNA in kidney tissue of rats in the control group were detected by Q-PCR. Increased expression of TNF-α, IL-6 and MCP-1 mRNA were observed and curcumin treatment significantly decreased expression of these markers. GAPDH was used as a housekeeping gene for normalization. Data are the means ± SD (n = 10). Statistical significance: *p < 0.05, **p < 0.01 versus the control group; ^#^p < 0.05, ^##^p < 0.01 versus the AKI and CO + AKI group, respectively.

To examine the protective effects of curcumin from inflammation and injury in the kidney, the expression levels of monocyte chemotactic protein-1 (MCP-1), TNF-α, IL-6 and the kidney injury marker genes, Kim-1 and Lipocalin-2 (Lcn-2) were assessed by quantitative polymerase chain reaction (q-PCR) (Fig. 5a). MCP-1, TNF-α, IL-1β, Kim-1 and Lcn-2 mRNA expression were all significantly up-regulated in AKI and CO + AKI groups compared with the control group (p < 0.01) and rats in these two groups displayed a similar levels of expression for all the markers tested (Fig. 5b). Treatment with curcumin decreased the expression levels of these genes in the CUR + AKI group compared with AKI and CO + AKI groups (p < 0.01 and p < 0.05, respectively).

### Effects of curcumin on apoptotic, oxidative stress and lipid oxidative signalling pathways in renal tissue

**Figure 6:**
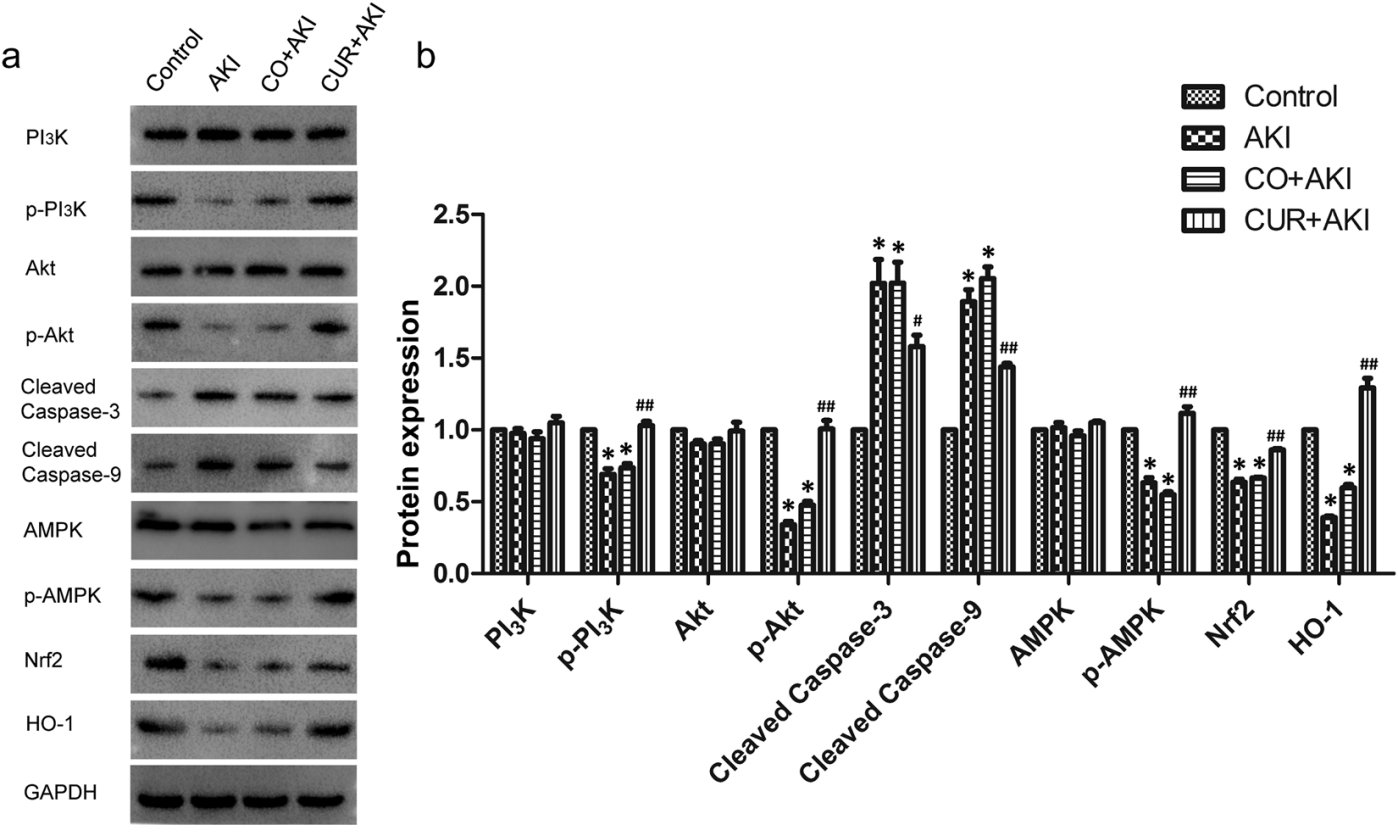
Effect of curcumin on apoptotic, oxidative stress and lipid oxidative signaling pathway associated proteins in kidney tissue. (**a**) Representative western blots depicting protein levels of PI_3_K, p-PI_3_K Akt, p-Akt, AMPK, p-AMPK, cleaved caspase-3, cleaved caspase-9, Nrf2 and HO-1 in kidney tissue. (**b**) The CUR + AKI group showed significantly increased levels of p-PI_3_K, p-Akt, p-AMPK, Nrf2, and HO-1, and decreased levels of cleaved caspase-3, cleaved caspase-9 compared to the AKI and CO + AKI groups. Data are the means ± SD (n = 10). Statistical significance: *p < 0.01 versus the control group; ^#^p < 0.05, ^##^p < 0.01 versus the AKI and CO + AKI group.

The potential mechanisms involved in the effects of curcumin on RM induced AKI was investigated. Protein expression of apoptotic-, oxidative stress- and lipid oxidative-related signaling pathway genes were detected by western blot (Fig. 6a). The expression of total PI_3_K, Akt, AMPK protein in each group was equal, while p-PI_3_K, p-Akt and p-AMPK were significantly lower in the AKI and CO + AKI groups compared with the control group (p < 0.01) (Fig. 6b), and was markedly up-regulated in the CUR + AKI group compared with the AKI and CO + AKI groups (p < 0.01). Down-regulation of caspase-3 and caspase-9 were observed in the CUR + AKI group compared with the AKI and CO + AKI groups (p < 0.05 and p < 0.01, respectively). Western blot analysis also showed that Nrf2 and HO-1 expression were decreased in AKI and CO + AKI groups compared with control group (p < 0.01), whereas treatment with curcumin significantly increased the expression of Nrf2 and HO-1 compared with rats in the AKI and CO + AKI groups (p < 0.01 for each).

## Discussion

AKI is characterized by a rapid decline of the glomerular filtration rate and retention of nitrogenous waste products. Traumatic and non-traumatic RM are now considered to be one of the leading causes of AKI^33^. RM is a syndrome characterized by skeletal muscle degeneration and muscle enzyme leakage causing high mortality^34^. The development of RM is associated with causes such as crush syndrome, earthquakes, exhaustive exercise, medications, infections and toxins^35, 36^.

The most commonly used model for studying this form of ARF is obtained in the rat by intramuscular injection of glycerol, which produces a myoglobinuric state similar to clinical RM^37^ and is characterised by rapid increases in BUN and Scr which are associated with a marked reduction in glomerular filtration rate within 3 h after glycerol administration^33^. Glycerol-induced AKI in rodents is mediated by renal ischemia and myoglobin nephrotoxicity^33, 38^. In glycerol-induced AKI, myoglobin heme induces oxidative stress and lipid peroxidation of the proximal tubular cells, triggering the release of a series of mediators, including cytokines and chemokines, leading to leukocyte activation and subsequent tubular necrosis in the renal cortical area^39–42^. The symptoms that follow intramuscular injection of glycerol in the rat mimics the AKI seen in crush injury, and renal histological changes such as tubule injury are similar to those observed clinically^43, 44^. Therefore, this animal model was used in this study to investigate the effects of curcumin against AKI induced glycerol injection.

Curcumin is an active ingredient of polyphenolic curcuminoids extracted from the species *Curcuma longa Linn*. Curcumin has been well-recognized as a dietary spice for centuries, and its pharmacological activity has been studied in various animal models and clinical investigations, which have demonstrated anti-inflammatory and antioxidant effects^45, 46^. This study showed that curcumin improved renal function in RM-induced AKI. We evaluated kidney injury 72 h, as AKI has previously been shown to be most severe at that time point^22^. Rats in the AKI group exhibited significantly increased BUN, Scr and CK levels as well as significant gross and micro-morphological changes. In contrast, rats that were given curcumin exhibited markedly lower BUN, Scr and CK levels, exhibited less renal tubular epithelial cell degeneration and necrosis, and had significantly improved tubule necrosis scores and ovrerall better cellular microstructure compared to AKI rats. However, there was no significant difference between rats in the AKI and CO + AKI groups. Therefore, in the current study, corn oil played a minimal role in glycerol-induced AKI.

Furthermore, curcumin increased serum SOD and GSH-Px, a superoxide radical scavenger enzyme and a peroxide decomposition enzyme, respectively, while at the same time, decreased urinary MDA, a lipid peroxidation index marker. These data indicated that antioxidant related pathways may be activated by curcumin, thus protecting renal function against AKI^47^. Immunohistochemical and western blot results also showed that curcumin significantly increased expression of HO-1 and Nrf2 and suggest that curcumin treatment protects the kidney from oxidative stress by activating regulating these two proteins. Nuclear factor erythroid 2-related factor 2 (Nrf2) regulates antioxidant genes including acting in synergy to remove reactive oxygen species (ROS) through sequential enzymatic reactions. It has been reported that curcumin can activate Nrf2 to up-regulate enzymes involved in antioxidant defense, like SOD and heme oxygenase-1 (HO-1)^48, 49^. In the present study, curcumin up-regulated HO-1 via activation of Nrf2, leading to lowered levels of MDA, and also increased ROS expression, which lowered SOD expression normally associated with AKI. Nrf2 binds to the antioxidant response elements (ARE) in the promoter regions of Nrf2 target genes in the nucleus, including HO-1^50^. The induction of HO-1 has generally been considered to be an adaptive cellular response to oxidative stress^51^. That Nrf2-mediated HO-1 induction contributes to antioxidant capacity has been demonstrated in multiple disease models, including cardiomyopathy in type 2 diabetic mice^52^, a rat model of transient global cerebral ischaemia^53^, and endotoxin shock-induced acute lung injury in rabbits^54^. Recently, Nrf2/HO-1 activation has also been shown to have protective effects in aristolochic-acid-induced AKI^55^ and type 2 diabetic nephropathy^29^, as well as intestinal ischemia–reperfusion induced acute renal injury^56^.

The expression of proliferating cell nuclear antigen (PCNA) and E-cadherin increased significantly in AKI rats treated with curcumin. PCNA is an auxiliary protein of DNA polymerase δ which plays a fundamental role in the initiation of cell proliferation. PCNA expression is an index of renal regeneration, as its level correlates directly with the rates of cellular proliferation and DNA synthesis^57^. E-cadherin is a cell adhesion molecule that plays an important role in maintaining renal epithelial polarity and integrity^22^. Thus, results indicate that curcumin can promote cell proliferation and kidney tissue recovery.

In our study, glycerol-induced AKI increased expression of TNF-α, IL-6, MCP-1, Kim-1 and Lcn-2, all which were significantly decreased after curcumin treatment. TNF-α and IL-6 are pro-inflammatory factors^58^ which are expressed in injury and inflammation sites that subsequently initiate the release of other pro-inflammatory cytokines and inflammatory mediatorse^59^. TNF-α also activates caspase-mediated apoptosis through activation of the death receptor pathway^55^. It was reported that TNF-α and IL-6 expression were significantly elevated in chronic renal failure patients^60^ and rat AKI models using cisplatin and paraquat to induce AKI^61, 62^. MCP-1 is a more recently studied renal biomarker which is expressed in injury and inflammation sites and directs the recruitment of macrophages, which bind to chemokine receptors to promote macrophage adhesion and chemotaxis^63^. Up-regulation of MCP-1 can occur in progressive kidney disease and when interstitial inflammatory infiltration occurs^63^ and due to this, the role of MCP-1 in AKI has attracted increasing attention^64^. In AKI, pro-inflammatory cytokines and chemokines are important factors that initiate inflammation and therefore play a prominent role in the progression of AKI^62^. The anti-inflammatory properties of curcumin in the kidney include the suppression of the pro-inflammatory factors nuclear factor (NF)-κB and cyclooxygenase (COX)-2, as well as the up-regulation of Nrf2 and HO-1^31, 32^. Kim-1 is a specific and sensitive biomarker of kidney injury^65, 66^ mRNA levels associated with this protein have been shown to increase more than any other gene after kidney injury. Furthermore, the ectodomain of Kim-1 has been shown to be cleaved from cells both *in vitro* and *in vivo* and deposited within the urine of rodents after proximal tubular injury^67^. Lcn-2, also named neutrophil gelatinase associated lipocalin, is a recently identified marker for acute kidney injury as well as chronic kidney disease^68–70^. Studies have shown that both Kim-1 and Lcn2 are induced in AKI animal models and human renal diseases that involve acute injury of the proximal tubule epithelium^71–73^.

Renal tubular epithelial cell apoptosis is important in the pathogenesis of RM-induced AKI. In the current study, TUNEL staining revealed that apoptotic cells in the kidneys of AKI rats treated with curcumin were markedly reduced. In addition, western blot results also showed that down-regulation of caspase-9 and caspase-3, as well as up-regulation of p-Akt were observed in the CUR + AKI group compared with the AKI and CO + AKI groups. These data suggested that curcumin treatment significantly reduce apoptosis through mediation of the PI_3_K/Akt signaling pathway, a cell survial pathway. We conclude that expression of phosphorylated PI_3_K and AKT may have inhibited the release of Bad and Bax, as well as other downstream pro-apoptotic protein caspases, resulting in the lowered levels of apoptotic cells in the CUR + AKI group^74–76^. Indeed, caspase-3 and caspase-9 are closely coupled to upstream, pro-apoptotic signals. Specifically, cleaved caspase-3 and cleaved caspase-9 are involved in a mitochondrial-dependent pathway that eventually leads to cell apoptosis^77^. In the present study, p-5′ adenosine monophosphate-activated protein kinase (p-AMPK) expression was significantly increased by curcumin treatment, which indicated that AMPK signaling pathway was activated in the CUR + AKI group. AMPK is considered a metabolic master switch, regulating several intracellular systems^78, 79^ and has recenty been identified as a regulator of renal hypertrophy in diabetes, a disease where it has been shown to be downregulated^80, 81^. Interestingly, several studies have revealed that AMPK signaling may play a specific role in renal cells^82, 83^. Our finding showed that activation of the AMPK signaling pathway may have produced synergistic effects with Nrf2/HO-1 signaling pathway on antioxidant responses in RM-induced AKI.

In conclusion, this study showed that curcumin exerted renoprotective effects by inhibiting oxidative stress in RM-induced AKI through activation of the AMPK and Nrf2/HO-1 signaling pathways and ameliorated RM-associated renal injury and cell apoptosis through activation the PI3K/Akt pathway. Taken together, these results indicate that curcumin might be a useful therapeutic agent for treatment of AKI, although further studie are warranted.

## Methods

### Chemicals

All chemicals and reagents were purchased from Sigma Chemical Co. (St. Louis, MO, USA) unless otherwise indicated.

### Animal Care and Use

All animal experiments were approved by the Institutional Animal Care and Use Committee (IACUC) of the Nanjing Medical University, Jiangsu, China, and methods were carried out in “accordance” with the approved guidelines. Animals were obtained from the Animal Core Facility of Nanjing Medical University, Nanjing. Sixty female Sprague Dawley (SD) rats weighting 200–250 g were housed in a 12 h dark/light cycle animal facility with controlled temperature (20–25 °C) and humidity (40–70%). Food and water were given *ad libitum* throughout the study.

### Experimental design

Sixty female SD rats were allowed to acclimatize for 1 week after which they were deprived of water for 24 h before the experiment and randomized into four groups. Rats in the control group received half the dose of saline (10 ml/kg) in each hind limb muscle; those in the group AKI received half the dose of glycerol (10 ml/kg, 50% v/v in sterile saline) in each hind limb muscle^22, 84^; rats in the CUR + AKI group received curcumin 200 mg/kg/day orally as a suspension in corn oil^25, 85, 86^ after glycerol administration; rats in the CO + AKI group received the same daily oral volume of corn oil as the group CUR + AKI. Three days after glycerol injection, all rats were sacrificed under general anesthesia using intraperitoneal injection of pentobarbital sodium (150 mg/kg). Blood was collected through heart puncture and the right kidney was excised immediately and divided into four parts. One part was fixed in glutaraldehyde solution for TEM examination and the other was stored in −80 °C for biochemical analysis. Finally, the left kidney was fixed in 10% neutral buffered formalin for histological studies.

### Sample preparation and biochemical assays

All blood samples were allowed to clot at room temperature and centrifuged at 2,000 *g* for 10 min to harvest serum. Serum biochemical parameters of BUN, and Scr and CK levels were measured (n = 12 per group) using the commercially available kits, BUN (995–17711), Scr (991–32593), and CK (994–64291), all from Wako Pure Chemical Industry, Japan.

The kidneys were excised, then washed in ice-cold saline and homogenized in 0.1 M Tris–HCl buffer (pH 7.4). The homogenate were first centrifuged at 10,000 *g* for 15 min and the supernatants were then centrifuged at 100,000 *g* for 1 h. The resulting supernatant (cytosolic fraction) was used for the determination of enzymatic activities and lipid peroxidation. Kidney biochemical parameters of SOD, MDA and GSH-Px were measured spectrophotometrically (Eon, BioTeK, USA) using the commercially available kits, SOD (A001-1), MDA (A003-1), and GSH-Px (A005), all from Jiancheng Bioengineering Institute, Nanjing, China.

### Histopathological examination

After sacrifice and dissection, kidneys from each rat were rapidly excised and then perfused in saline solution. Kidneys of rats from the different groups were fixed in 10% neutral buffered formalin for 24 h, dehydrated in graded alcohol and embedded in paraffin. The paraffin embedded tissues were then cut into 5-μm thick sections and stained with hematoxylin and eosin (H&E). Renal tubule injury was scored semi-quantitatively according to a scoring system described previously^87, 88^. Briefly, renal injury was defined by necrotic lysis, tubule dilation, cast formation, sloughing of cellular debris into the tubule lumen, or naked tubule basement membrane. Tubules in the boundary area between the renal cortex and medulla, and in the outer strip of the outer medulla were included. Renal injury scores were determined by the percentage of tubules injured: 0, no injury; 1, <20%; 2, 21–50%; 3, >50%; and 4, total destruction of all epithelial cells. Five random fields for each kidney slide (Five slides per animal) were examined (n = 8 per group).

### Immunohistochemical staining

For immunohistochemical analysis, the paraffin sections were first dewaxed after which heat-mediated antigen retrieval was performed by microwaving sections for 20 min in 10 mM sodium citrate, pH 6.0. Sections were allowed to cool for 15 min, followed by a brief wash in deionized water, and then rinsed twice in PBS. Sections were incubated for 30 min in 5% goat serum in DPBS containing 0.1% Tween and 0.5% BSA. The sections were incubated overnight at 4 °C with primary antibody PCNA (sc-56), HO-1 (sc-390991) and E-Cadherin (sc-71007); all from Santa Cruz Biotechnology, CA, USA, at the appropriate dilution. The secondary antibody Dako REAL EnVisio Detection System (K5007, DAKO, Denmark) was used to detect primary antibodies. The specimens were then counterstained with hematoxylin for 1 min. All sections were incubated under the same conditions with the same concentration of antibodies and at the same time, in order to have comparable immunostaining among the different experimental groups. Tissue sections in the boundary area between the renal cortex and medulla were observed and photographed with a microscope and were semi-quantified by Image-Pro Plus 6.0 software. The integrated optical density (IOD) of each photograph was collected. Five fields of each slice (Five slides per animal) were randomly selected for blinded measuring (n = 8 per group). Images were quantified by the immunoreactive area (IA) in μm^2^ and the IOD. Staining intensity (SI) for each image was calculated as SI = IOD/IA and mean with standard deviation was obtained for each series.

### TEM examination

Each specimen was prepared at all steps with an electron microscope reagent to generate ultrathin sections, and examined under a transmission electron microscope (Tecnai G2 Spirit Bio TWIN, FEI Ltd., USA). Electron micrographs of five to ten glomeruli per kidney were randomly examined for each experiment (n = 6 per group).

### *In situ* TUNEL fluorescence staining assay

The terminal deoxynucleotidyl transferase-mediated deoxyuridine triphosphate nick end labeling (TUNEL) assay was performed according to the manufacturer’s instructions (11684817910, Roche, Switzerland). Kidney tissue was fixed in 10% formalin overnight, dehydrated, embedded in paraffin, cut into 4 µm-thick sections, and placed on a numbered polylysine-coated glass slide. TUNEL-positive cells, which were stained green; nuclei were stained with DAPI to observe the nature of the TUNEL-positive cells. TUNEL-positive cells and total cells in the boundary area between the renal cortex and medulla were counted and analyzed using Image-Pro Plus 6.0 software. Five random fields per slide (Five slides per animal) were examined in each experiment (n = 8 per group).

### Q-PCR

Total mRNA was extracted from renal cortical tissue sample using TRIzol Reagent (B5704–1, Takara, Dalian, China) according to the manufacturer’s instructions, followed by treatment with DNase I (2212, Takara) according to the manufacturer’s instructions. RNA quality and quantity were determined using a Spectro-photometer (NanoDrop 2000c, Thermo Scientific, USA) and cDNA was synthesized immediately after using PrimeScriptTM RT reagent Kit (RR037A, Takara, Dalian, China), according to the manufacturer’s instructions. Q-PCR was performed using the Light Cycler PCR QC Kit (Roche, Switzerland) and the 7300 Real-Time PCR System (LC96, Roche). PCR primers are listed in Supplementary Table 1. Data analysis was performed using GraphPad Prism 5 software (n = 10 per group).

### Western Blot

Western blotting was performed according to methods previously described^22^. Briefly, 50 ug of total lysate from kidney tissue were subjected to 15% polyacrylamide gel electrophoresis and transferred to cellulose acetate membranes. The membranes were blocked with 1× casein solution for approximately 4 h and then incubated with rabbit monoclonal anti-P-AMPK (Ser485) (2537), anti-P-Akt (Ser473) (4058), anti-Akt (4685), anti-cleaved caspase-3 (Asp175) (9664) and anti-Cleaved Caspase-9 (Asp330) (7237), all from Cell signaling technology; or rabbit polyclonal anti-Nrf2 (sc-722), anti-GAPDH (H-12) (sc-166574) and mouse monoclonal anti-HO-1 (sc-390991) anti-AMPK (sc-398861), all from Santa Cruz Biotechnology; or PI3K (ab86714) and p-PI3K (ab182651), both from Abcam Biotechnology, in blocking buffer for 2 h at room temperature. Membranes were then washed three times with Tris-buffered saline-Tween-20 (TBST), incubated with goat anti-rabbit IgG-HRP secondary antibody (sc-2004, Santa Cruz Biotechnology) and imaged by ChemiDoc XRS + Molecular Imager (Bio-Rad, USA). All images were using the free image analyzing software Image J 1.42 (n = 10 per group).

### Statistical analysis

Statistical analysis was performed using SPSS v.16. Results values are means ± standard error (SE) and all statistical comparisons were made by means of one-way ANOVA test followed by Duncan’s multiple range test post hoc analysis. Student’s t-test was used to compare differences between means. p values of <0.05 were considered significant.

## Electronic supplementary material

Supplementary information for Effect of curcumin on glycerol-induced acute kidney injury in rats
