# Supplementary material for: Effect of curcumin on glycerol-induced acute kidney injury in rats
Source: Sci Rep. 2017 Aug 31;7:10114. doi: 10.1038/s41598-017-10693-4 (PMC5579036; doi:10.1038/s41598-017-10693-4)
Supplement: Supplementary file 1 — Supplementary information for Effect of curcumin on glycerol-induced acute kidney injury in rats [file 41598_2017_10693_MOESM1_ESM.doc]

**Supplementary information for**

**Effect of curcumin on glycerol-induced acute kidney injury in rats**

Jindao Wu^1^*, Xiongxiong Pan^2^*, Heling Fu^3^, Yuan Zheng^3^, Youjin Dai^3^, Yuan Yin^3^, Qin Chen^3^, Qingting Hao^3^, Dan Bao^3^ & Daorong Hou^3^

*^1^ Key laboratory of Living Donor Liver Transplantation; National Health and Family Planning Commision; Department of Liver Transplantation Center; The first Affiliated Hospital of Nanjing Medical University, 300 Guangzhou Road, Nanjing, 210029, People’s Republic of China.*

*^2^ Department of Anesthesiology, First Affiliated Hospital of Nanjing Medical University, 300 Guangzhou Road, Nanjing, 210029, People’s Republic of China.*

*^3^ Animal Core Facility of Nanjing Medical University, Nanjing Medical University, 101 Longmian Avenue, Nanjing, 211166, People’s Republic of China.*

** Jindao Wu and Xiongxiong Pan contributed equally to this work.*

**Supplementary Figures**

**Supplementary Figure 1.** Renal injury scoring and quantitative analysis of rat kidney from control group, AKI group, CO+AKI group and CUR+AKI group. Each bar represents the mean ± SD (n = 8). Statistical significance: * p < 0.01 versus the control group; ^#^ p < 0.01 versus the AKI and CO+AKI group.

**
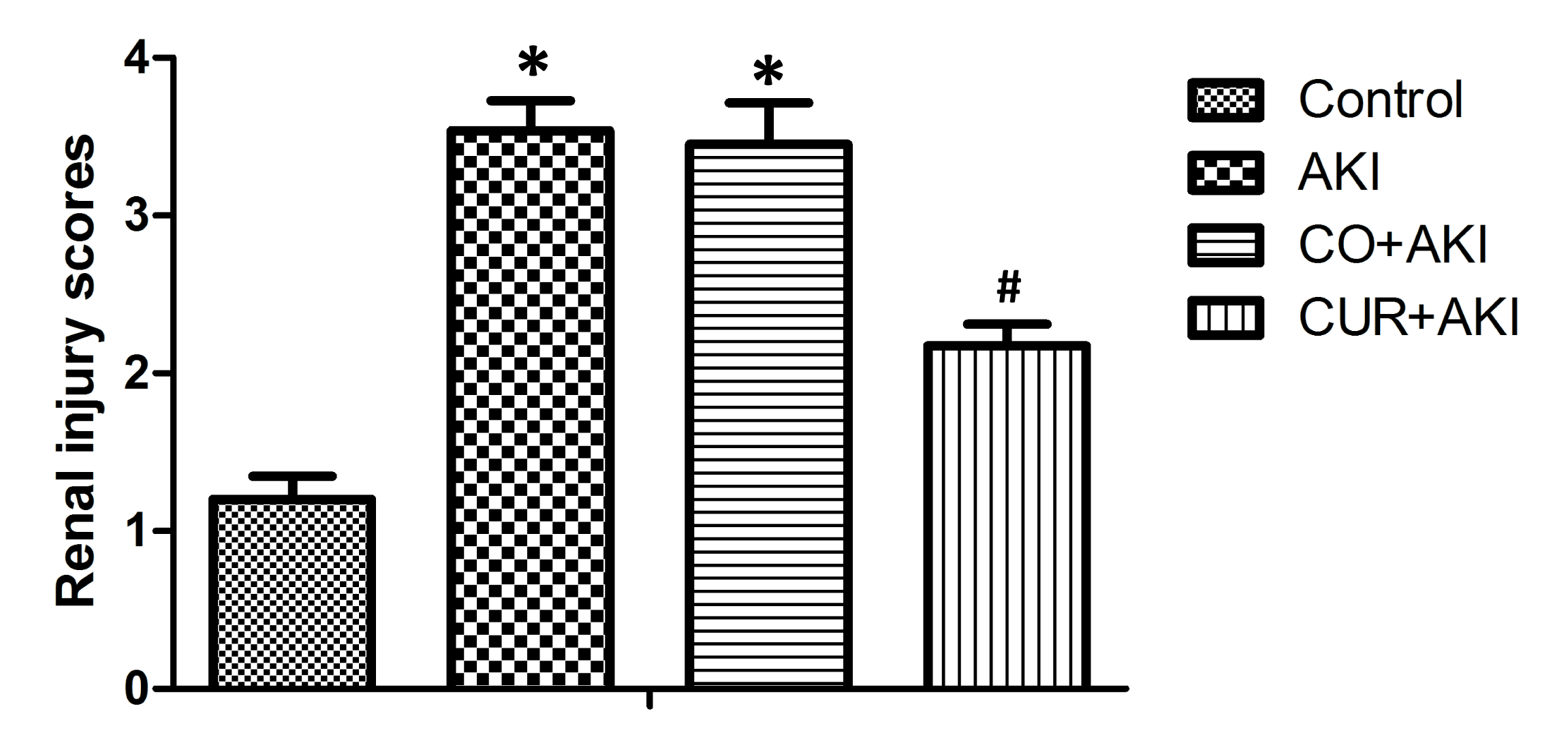
**

**Supplementary Figure 2.** (a) Additional figures of immunohistochemical staining of PCNA, HO-1 and E-cadherin in sections of rat kidney from control group, AKI group, CO+AKI group and CUR+AKI group. (b) Semi-quantitative evaluation of PCNA, HO-1 and E-cadherin expression represented as IOD/μm^2^. Each bar represents mean ± SD (n=6). Statistical significance: * p < 0.01 versus the control group; # p < 0.01 versus the AKI and CO+AKI group. IOD: integrated optical density.

**Supplementary Figure 3.** (a) Additional figures of in situ TUNEL fluorescence staining in sections of rat kidney from control group, AKI group, CO+AKI group and CUR+AKI group. (b) The levels of apoptosis were indicated as the percentage of TUNEL-positive cells among total cells. Data is the mean ± SD. Statistical significance: ***** p < 0.01 versus the control group; **^#^** p < 0.01 versus the AKI and CO+AKI group (n = 6).

**
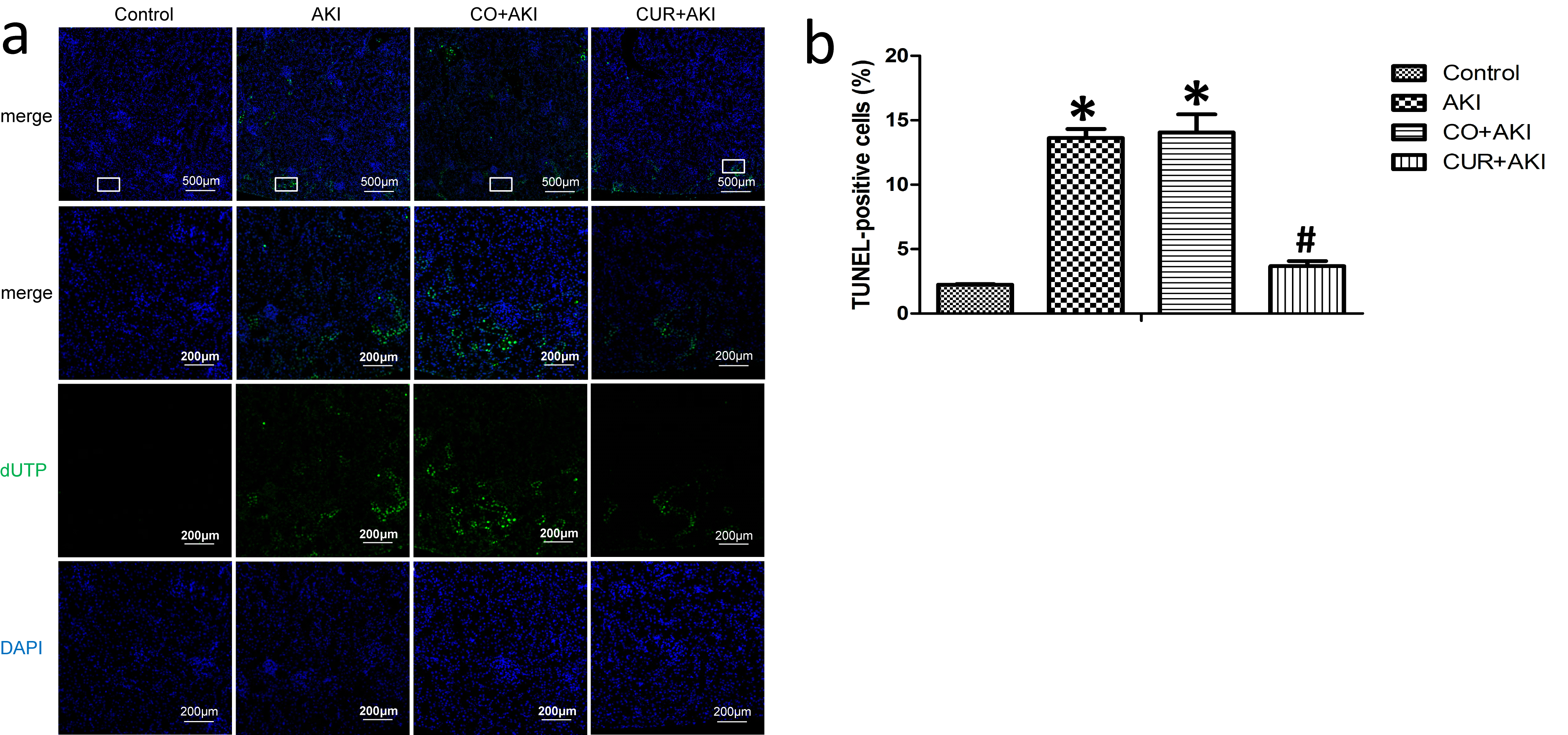
**

**Supplementary Figure 4.** Additional raw images of the Western blots shown in Figure 4. From left to right: Control group, AKI group, CO+AKI group, CUR+AKI group.


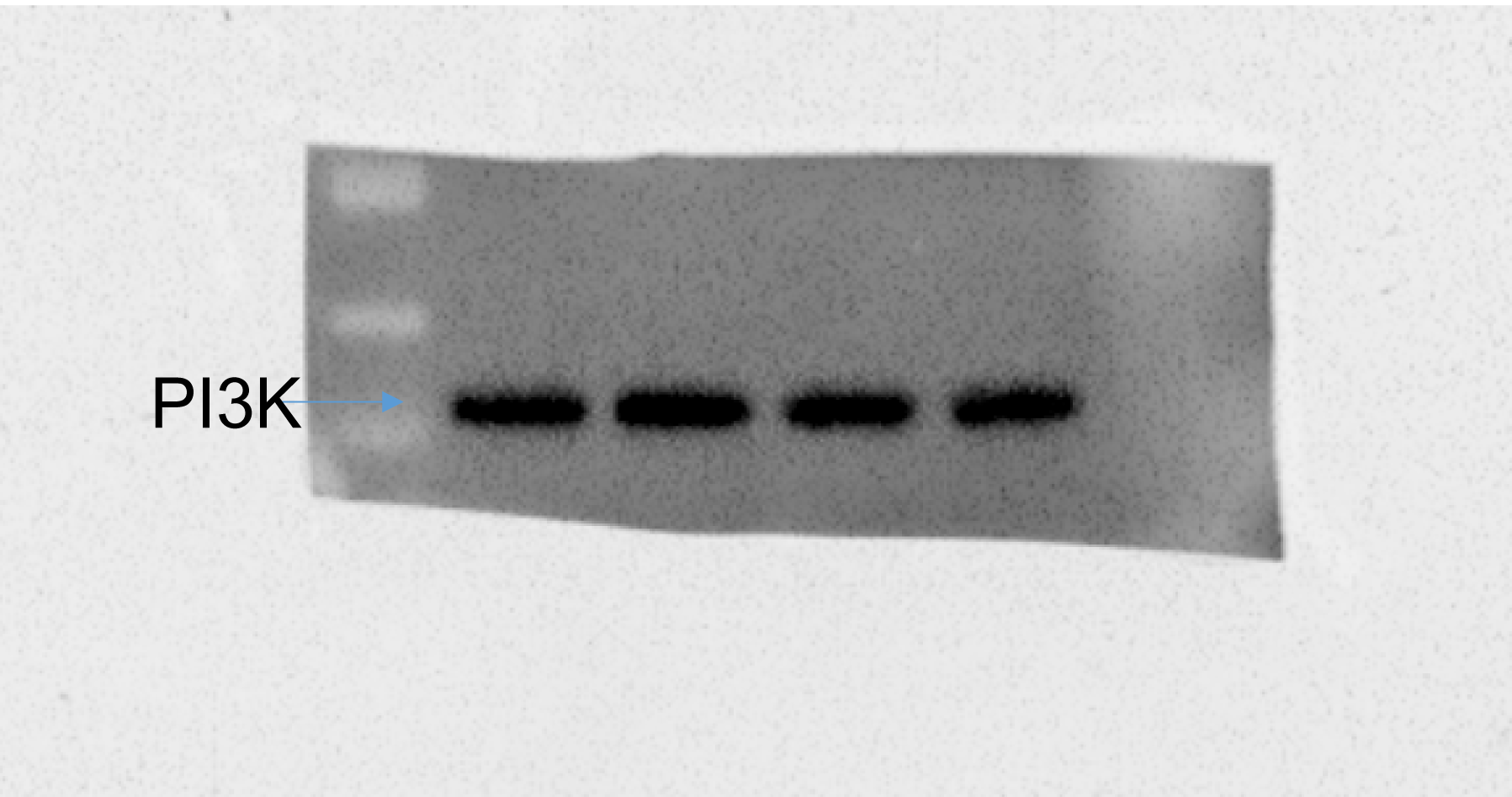


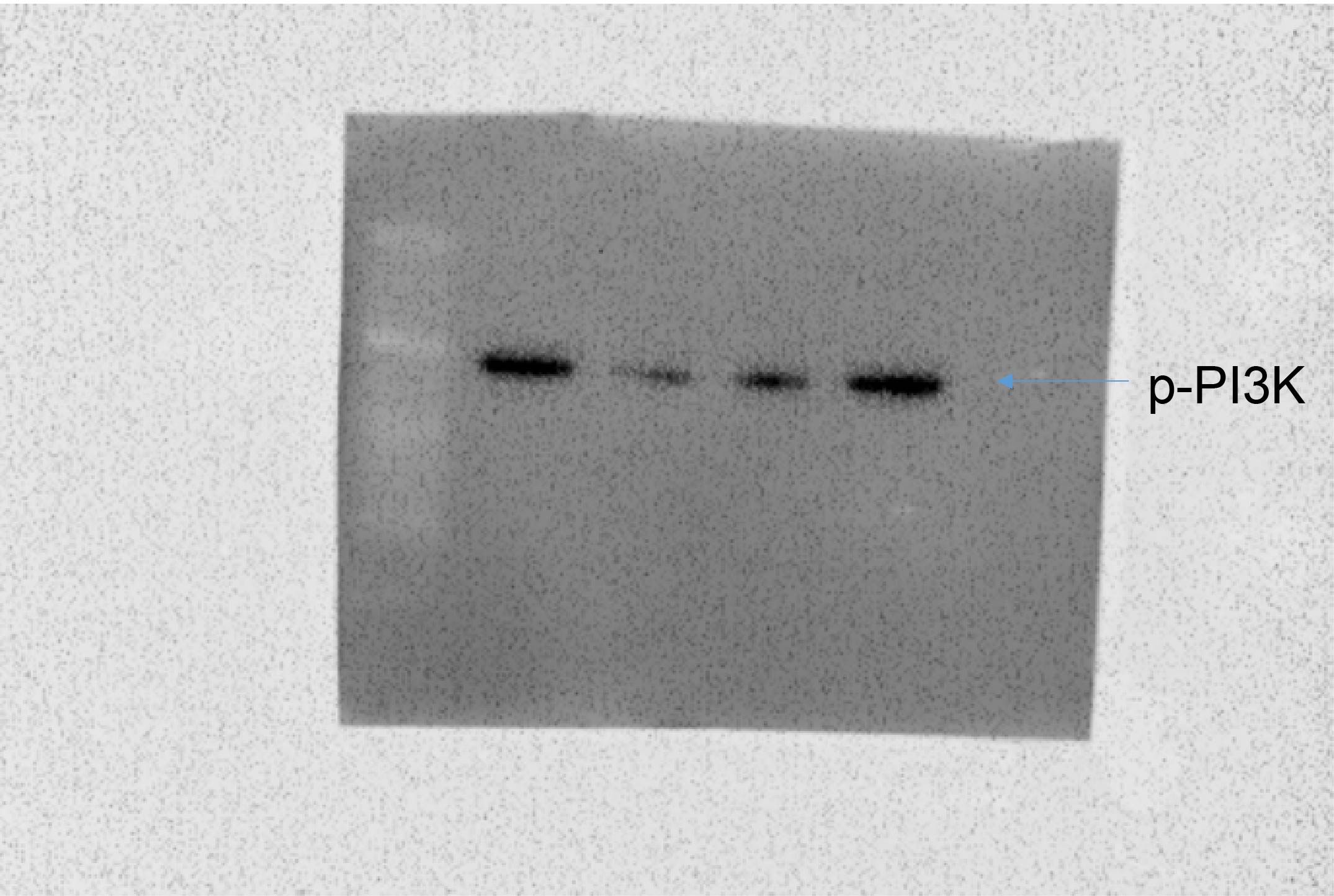


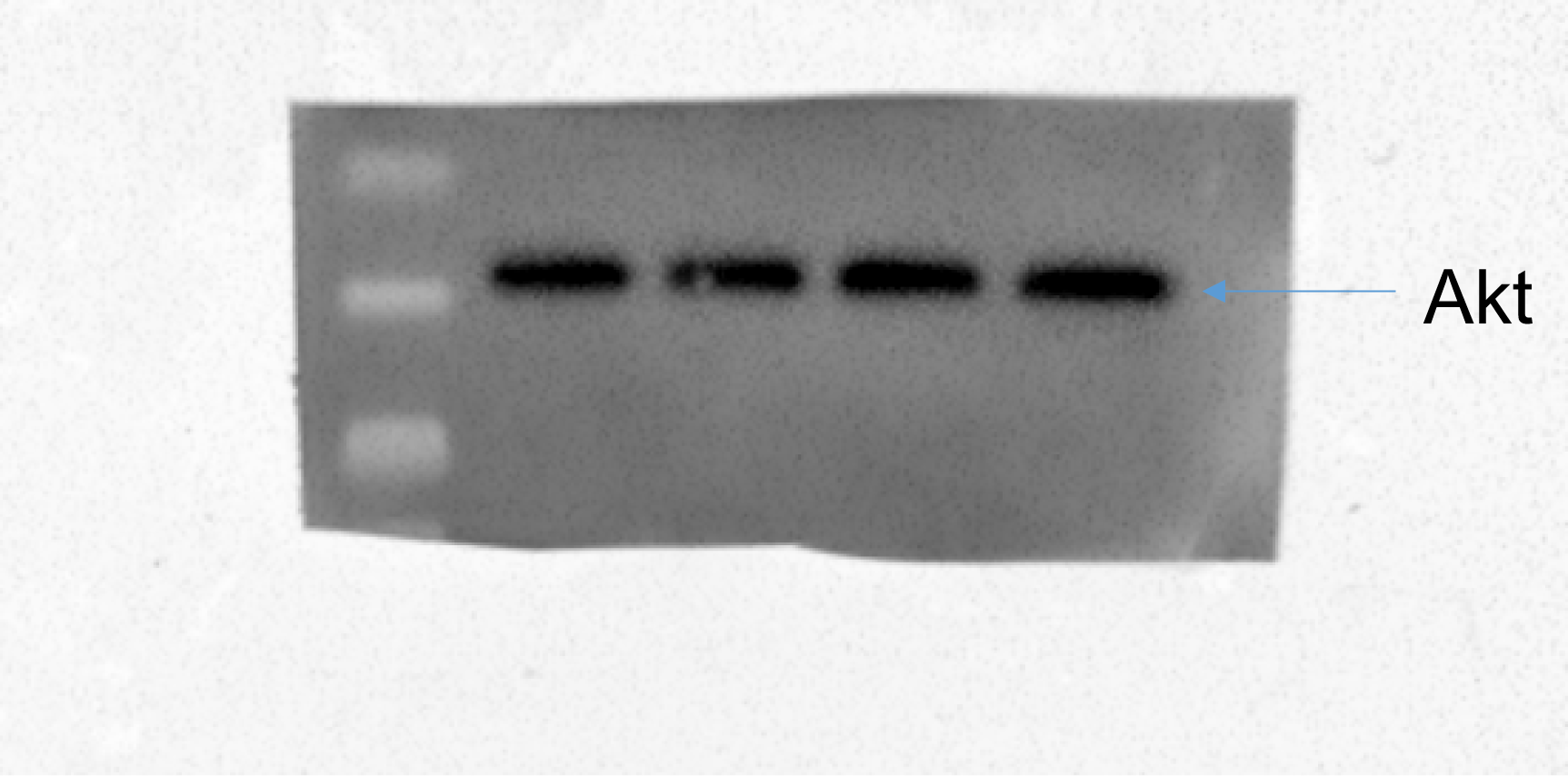


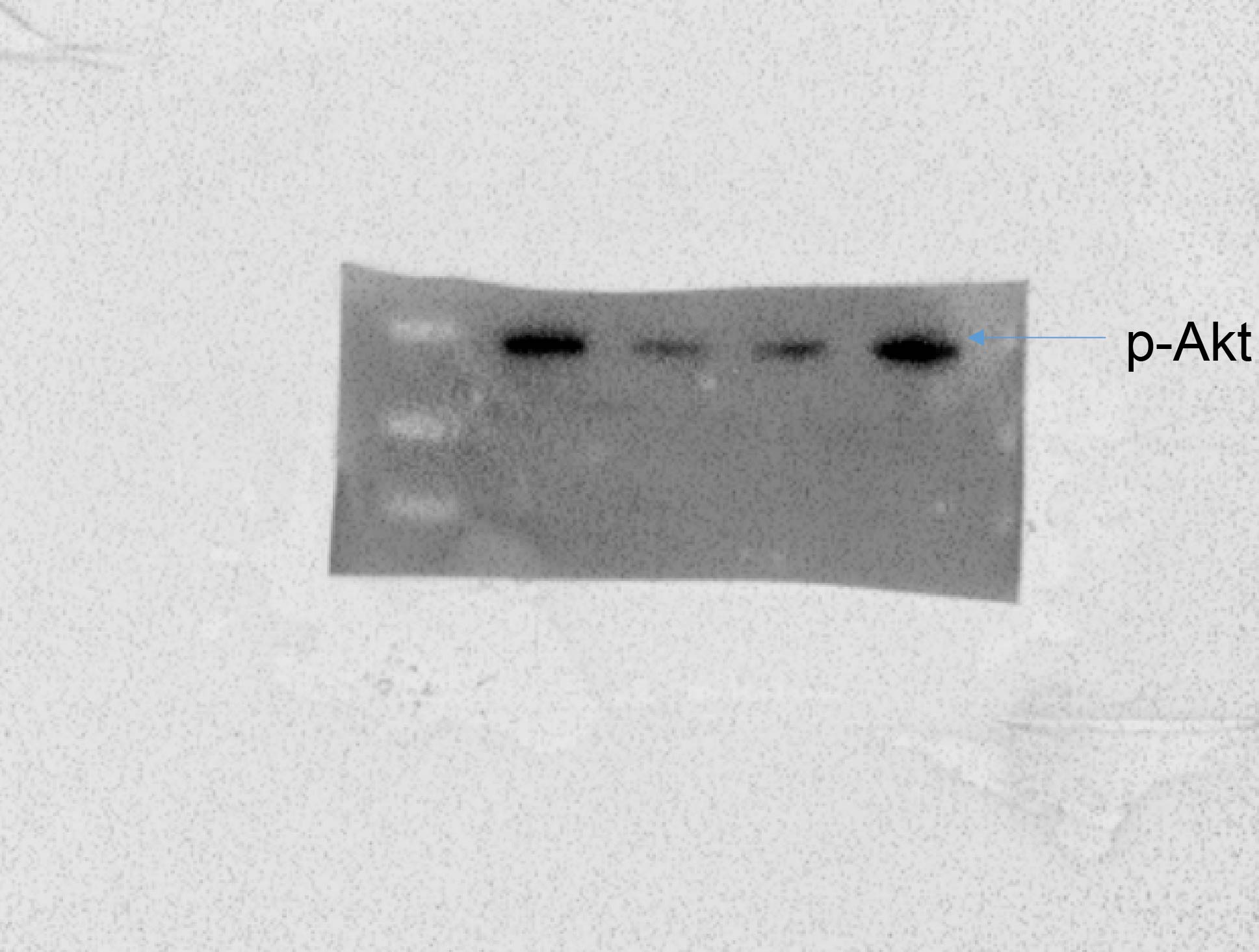


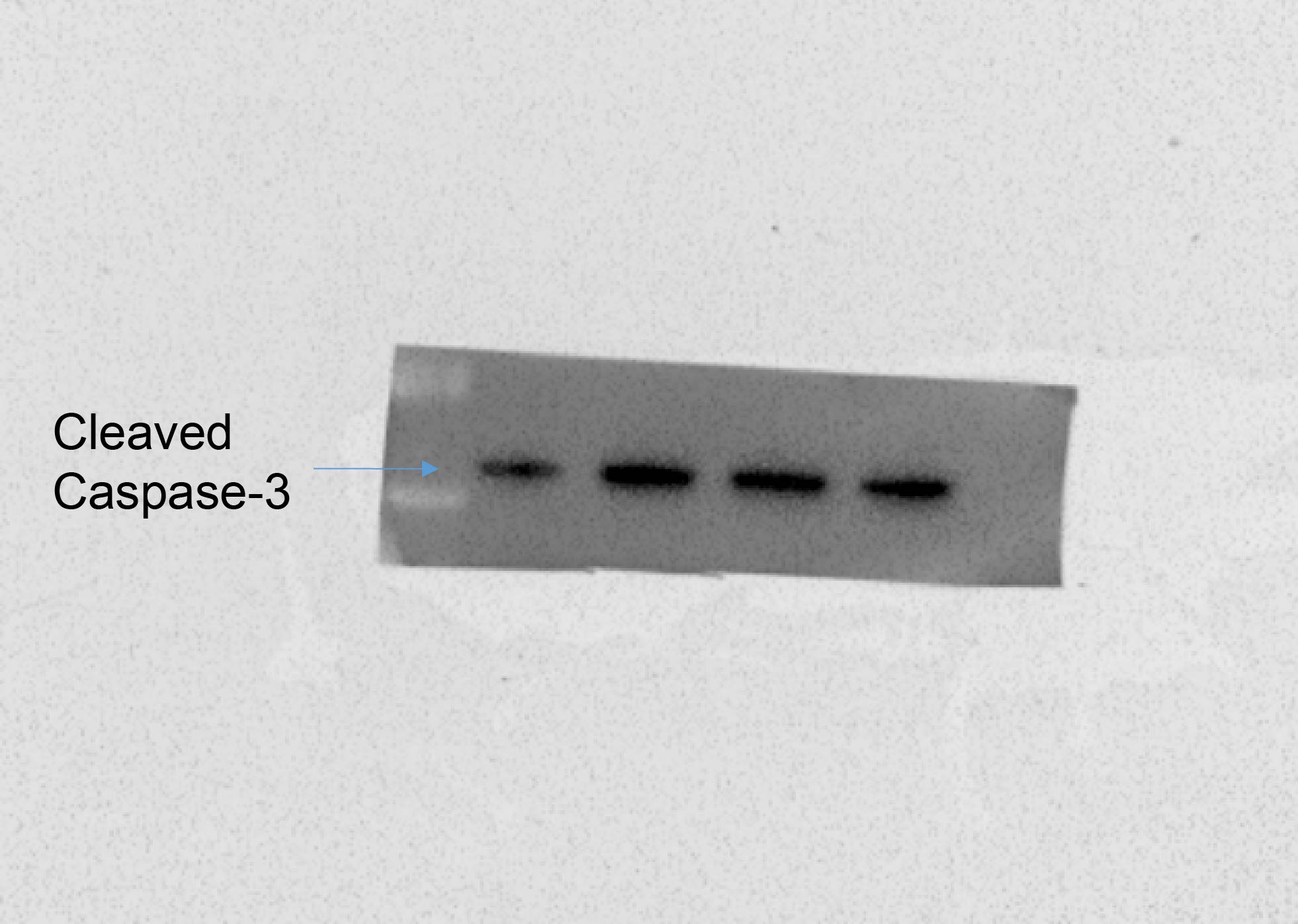


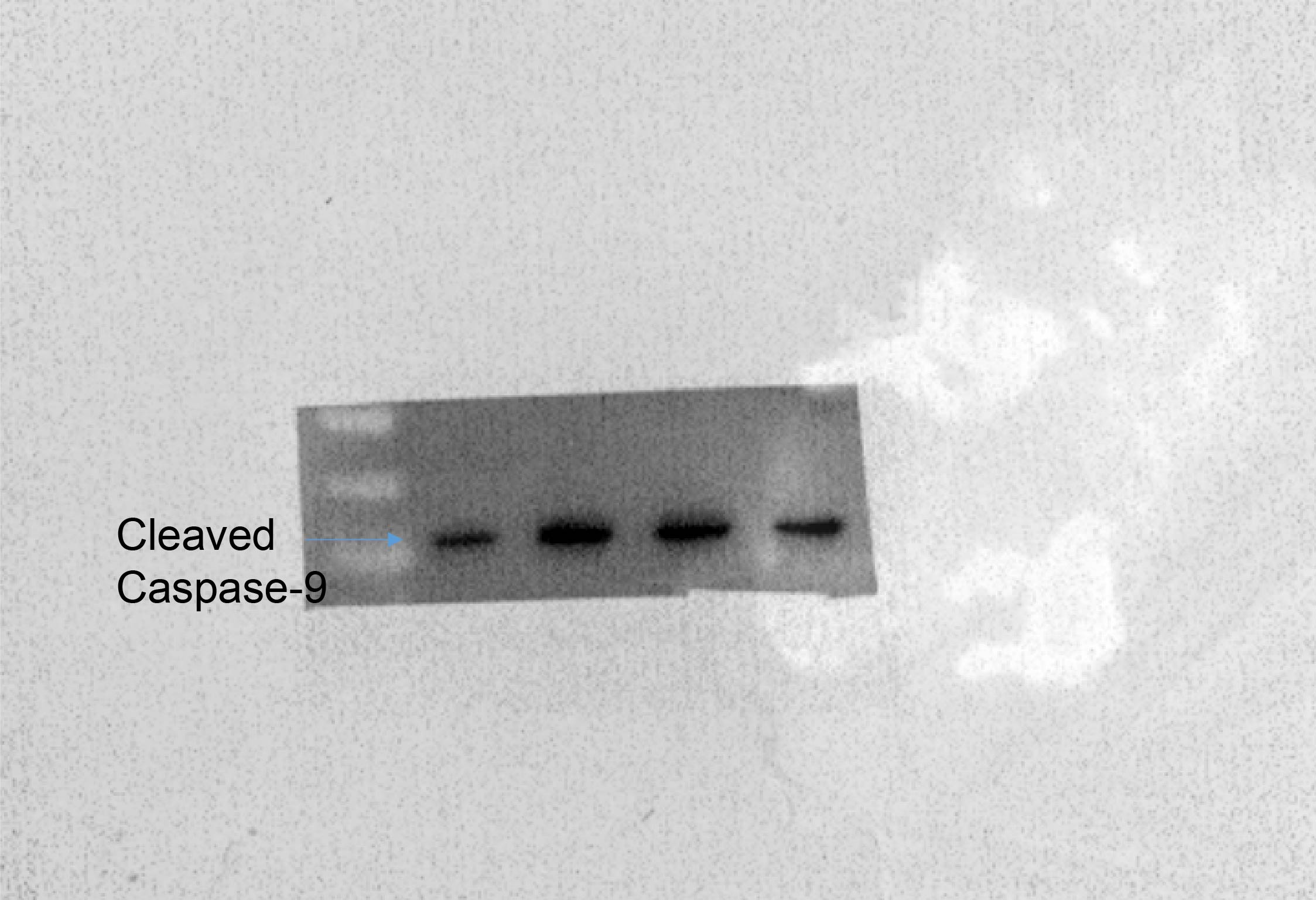


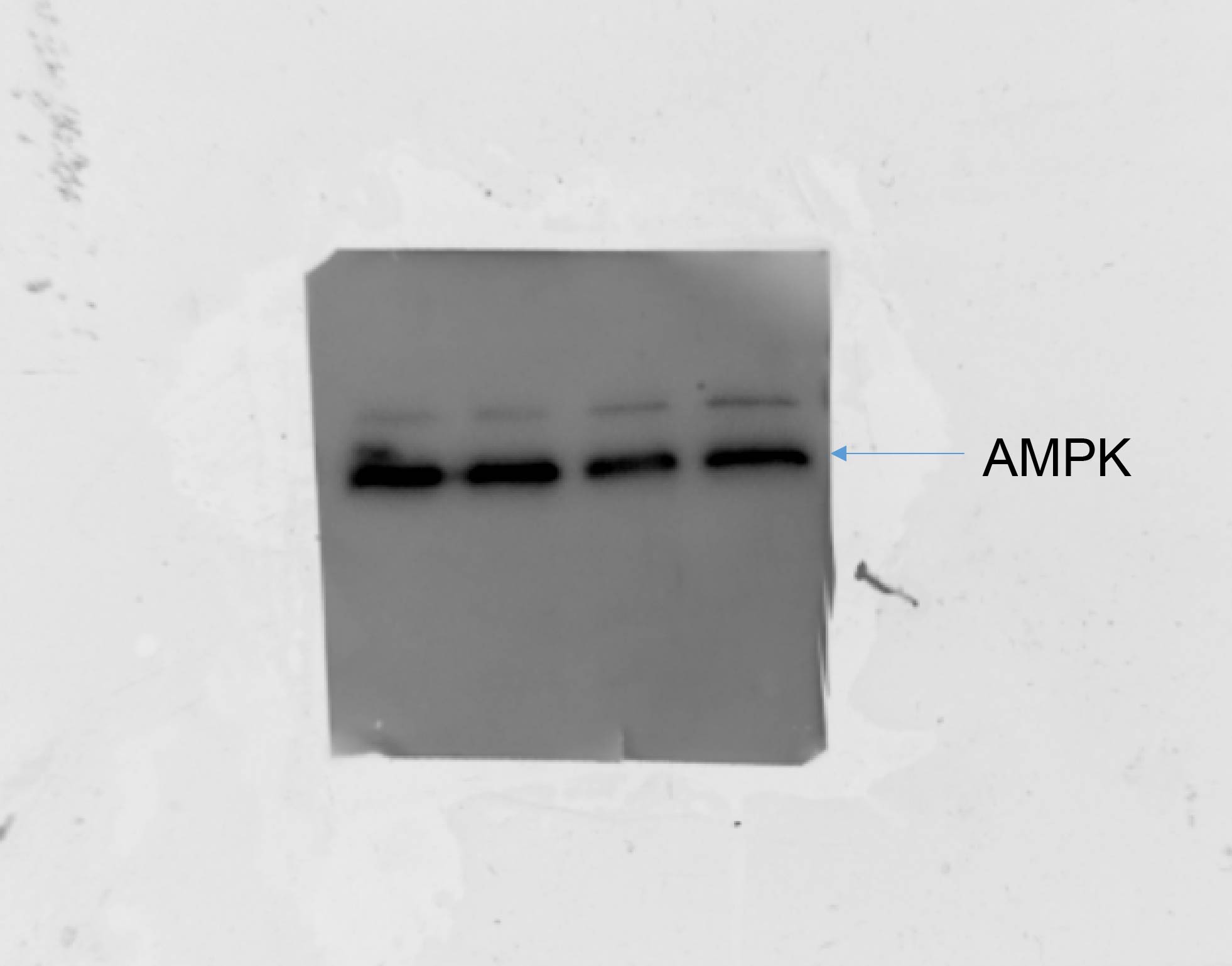


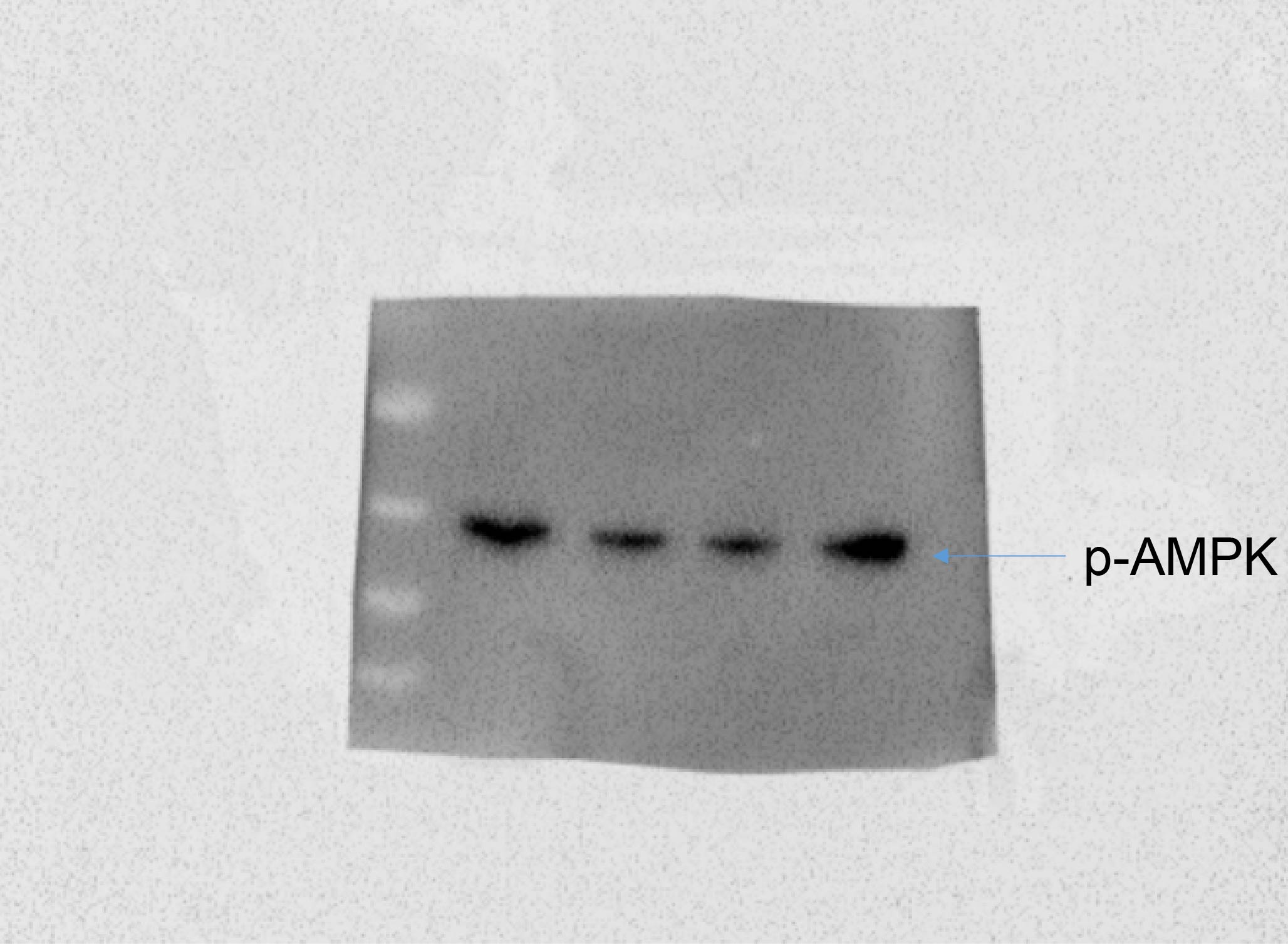


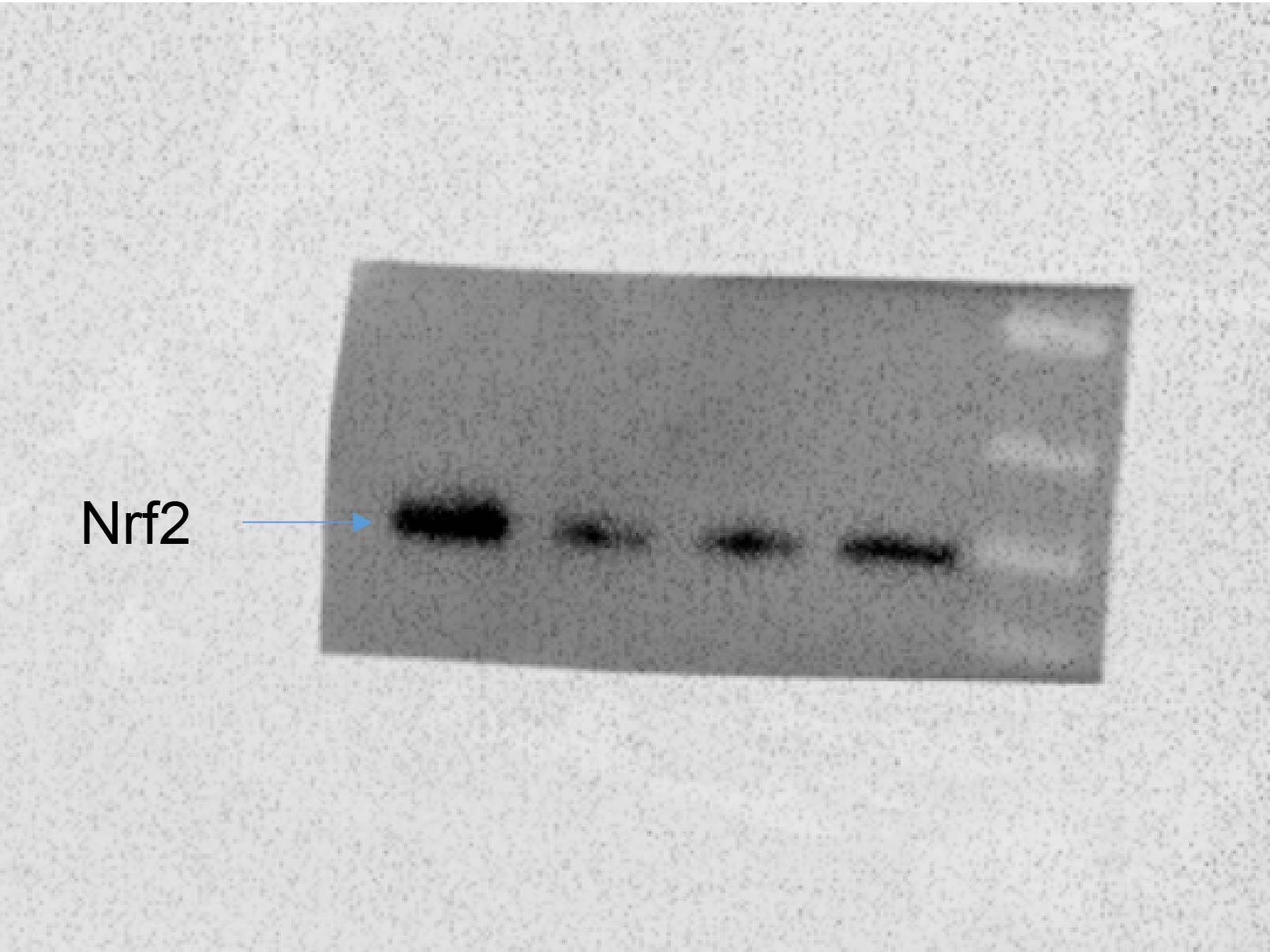


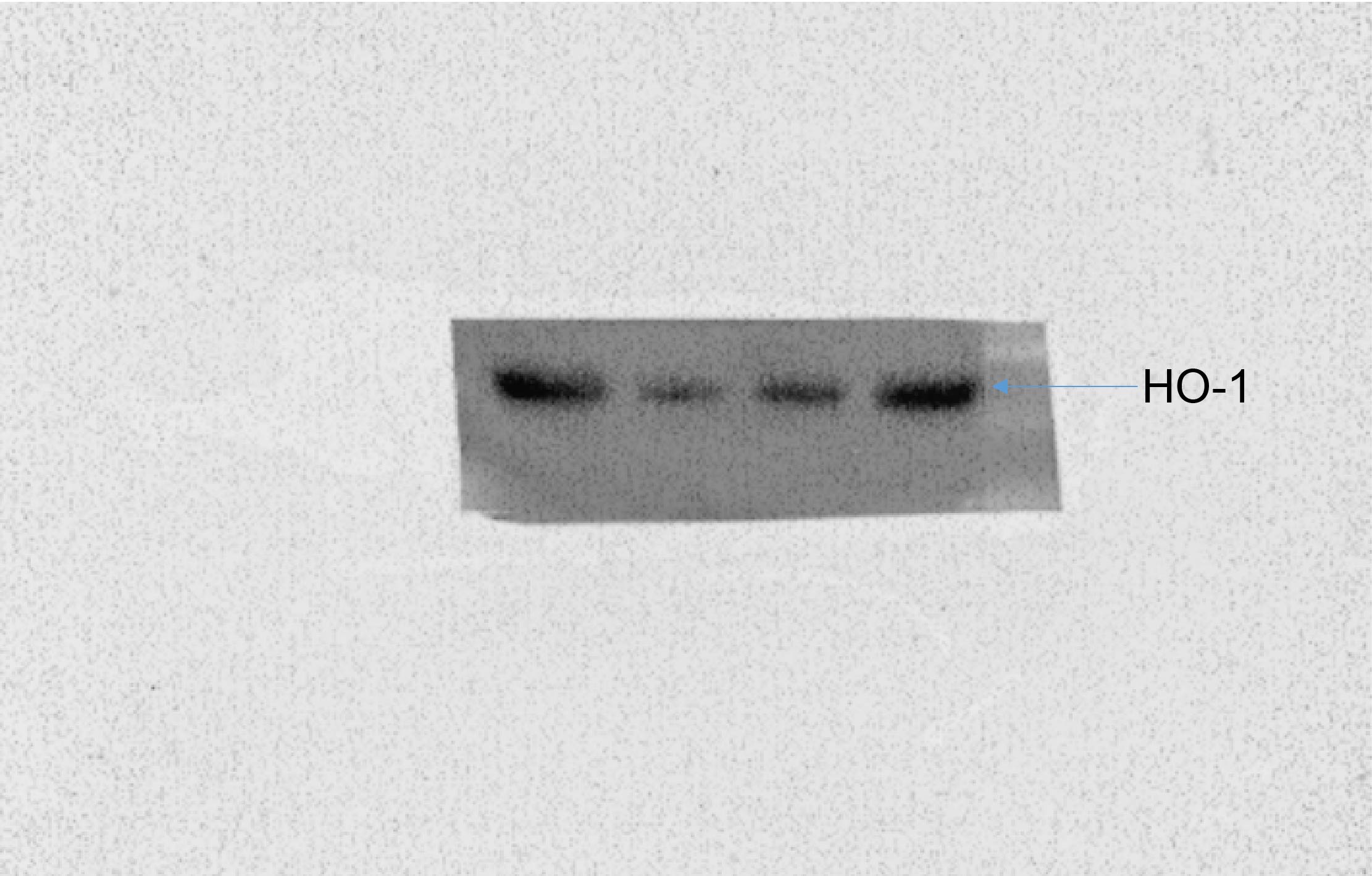


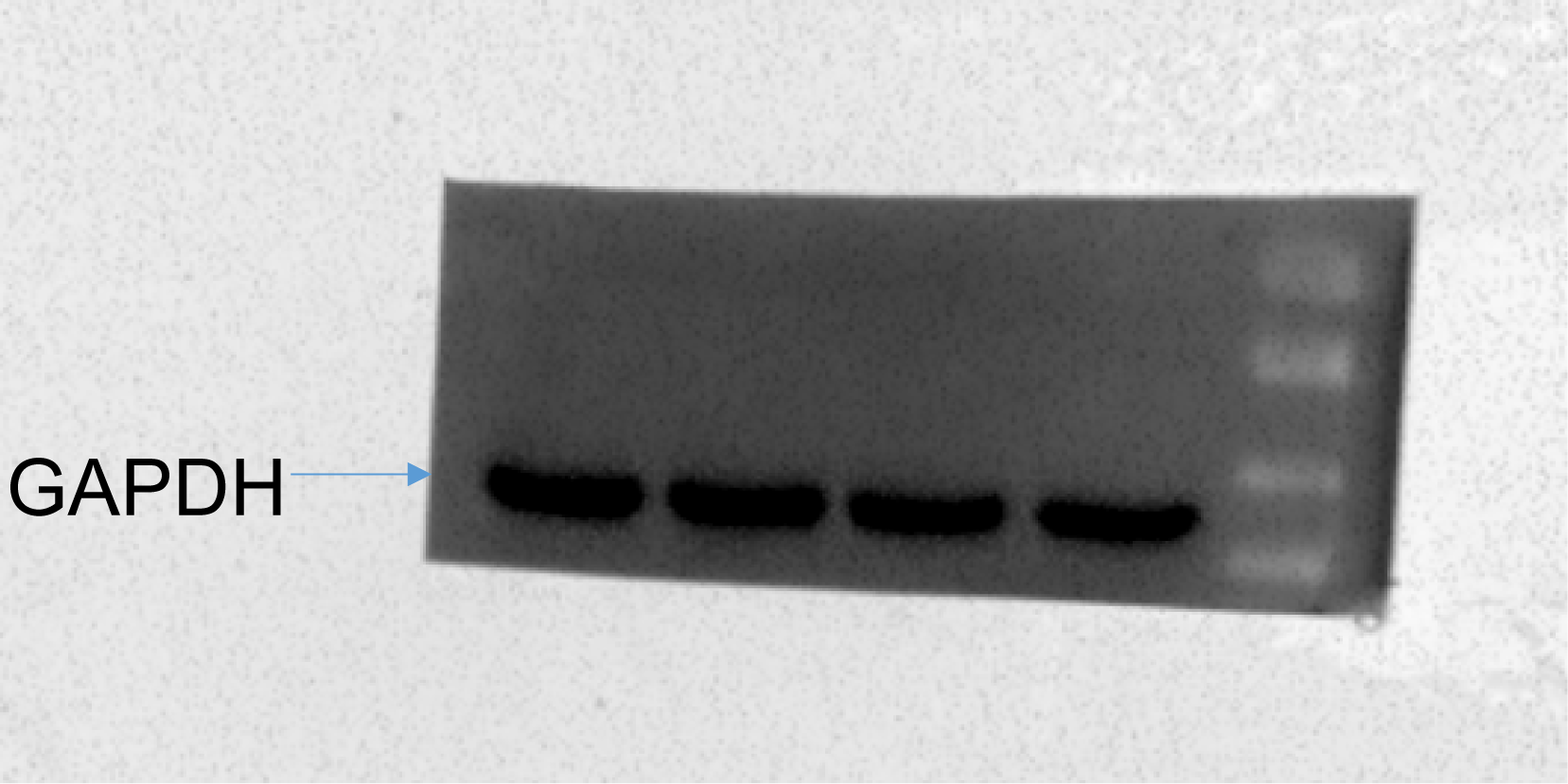


**Supplementary Table**

**Supplementary Table 1. List of primers and conditions for Q-PCR.**

| Genes | Primer sequence (5’-3’) | Accession Number | Products  size（bp） | Annealing  Temperature (℃) |
| --- | --- | --- | --- | --- |
| *TNF-α* | 5'-GTAGCAAACCACCAAGCG-3'  5'-GGTATGAAATGGCAAATCG-3' | NM_012675 | 211 | 54.5 |
| *IL-6* | 5'-CCACTGCCTTCCCTACTT-3'  5'-CATCATCGCTGTTCATAC-3' | NM_012589 | 180 | 48.5 |
| *MCP-1* | 5'-TGAACTTGACCCATAAATC-3'  5'-TGGAAGGGAATAGTGTAAT-3' | M57441 | 171 | 46.5 |
| *Kim-1* | TGGAGATTCCTGGATGGT  GAGGTGGAGACTCTGGTTGA | AF035963 | 175 | 50.5 |
| *Lipocalin-2* | CTGACTACGACCAGTTTGC  CTGACGAATCGCTCCTT | NM_130741 | 124 | 49.5 |
| *GAPDH* | 5’-CCATTCTTCCACCTTTGAT-3’  5’-TGGTCCAGGGTTTCTTACT-3’ | NM_017008 | 153 | 51 |
